# Characterization of moderate ash‐and‐gas explosions at Santiaguito volcano, Guatemala, from infrasound waveform inversion and thermal infrared measurements

**DOI:** 10.1002/2016GL069098

**Published:** 2016-06-27

**Authors:** S. De Angelis, O. D. Lamb, A. Lamur, A. J. Hornby, F. W. von Aulock, G. Chigna, Y. Lavallée, A. Rietbrock

**Affiliations:** ^1^ School of Ocean and Environmental SciencesUniversity of LiverpoolLiverpoolUK; ^2^ Instituto Nacional de Sismología, VulcanologíaMeteorología e Hidrología (INSIVUMEH)Guatemala CityGuatemala

**Keywords:** volcano infrasound, thermal infrared, Santiaguito

## Abstract

The rapid discharge of gas and rock fragments during volcanic eruptions generates acoustic infrasound. Here we present results from the inversion of infrasound signals associated with small and moderate gas‐and‐ash explosions at Santiaguito volcano, Guatemala, to retrieve the time history of mass eruption rate at the vent. Acoustic waveform inversion is complemented by analyses of thermal infrared imagery to constrain the volume and rise dynamics of the eruption plume. Finally, we combine results from the two methods in order to assess the bulk density of the erupted mixture, constrain the timing of the transition from a momentum‐driven jet to a buoyant plume, and to evaluate the relative volume fractions of ash and gas during the initial thrust phase. Our results demonstrate that eruptive plumes associated with small‐to‐moderate size explosions at Santiaguito only carry minor fractions of ash, suggesting that these events may not involve extensive magma fragmentation in the conduit.

## Key Points

- Infrasound waveform inversion allows assessing source parameters of volcanic explosions
- Thermal infrared imagery allows investigating the transition from an initial gas thrust phase to a buoyant plume during volcanic eruptions
- Eruption plumes produced by small‐to‐moderate eruptions at Santiaguito are gas rich

## 1 Introduction

Volcanoes are efficient sources of infrasonic acoustic waves with frequencies below 20 Hz [*Johnson et al.*, 2004; *Fee and Matoza*, 2013; *Garcés et al.*, 2013]. The sudden release of hot gases and pyroclasts from volcanic vents generates infrasound via volumetric acceleration of the atmosphere. Over short source‐receiver distances, acoustic wave propagation in the atmosphere is characterized by a relatively simple Green's function. Therefore, at local distances from the source (<5 km) the effects of scattering have frequently been neglected and infrasound used to investigate the dynamics of volcanic explosions and their source mechanisms [*Kim et al.*, 2012]. Acoustic sources with dimensions significantly smaller than the characteristic wavelength of infrasound can be considered acoustically compact and, thus, approximated using multipole expansion [*Rossing*, 2016]. Several authors used infrasound to assess the strength of volcanic explosions measuring either gas velocity or volume flux at the vent. *Johnson* [2007] and *Johnson and Miller* [2014] described explosions at Karymsky and Sakurajima volcanoes as monopoles, which is an isotropically expanding source causing volumetric acceleration of the atmosphere and radiating infrasound uniformly in all directions; *Delle Donne and Ripepe* [2012] demonstrated the use of a monopole source model to assess the volumetric flux during explosions at Stromboli volcano. A dipole source model (i.e., two monopoles of equal strength and opposite phase, separated by a small distance) was used to evaluate gas velocity at the vent for eruptions at Shishaldin [*Vergniolle and Caplan‐Auerbach*, 2004], Augustine [*Caplan‐Auerbach et al.*, 2010], Eyjafjallajökull [*Ripepe et al.*, 2013], and Redoubt [*Lamb et al.*, 2015] volcanoes. *Matoza et al.* [2009] argued that the infrasound wavefield produced by sustained eruptions is best described by jet noise models, and thus, an acoustic quadrupole may represent a more accurate representation of the radiation mechanisms at the source. *Kim et al.* [2012] recently proposed a waveform inversion workflow based on a representation of the acoustic source as a combination of multipole terms, using analytical Greens functions in a half‐space. This method can simultaneously retrieve the time history of mass outflow at the vent and account for directivity effects on the acoustic wavefield. In this study, we applied the waveform inversion method of *Kim et al.* [2012] to infrasound signals from small and moderate gas‐and‐ash explosions at the Santiaguito lava dome complex, Guatemala. For one explosion, we complemented infrasound with the analysis of thermal infrared data and measurements of ash density to constrain the relative volume fractions of gas and ash in the eruptive plume.

## 2 Activity at Santiaguito

**Figure 1:**
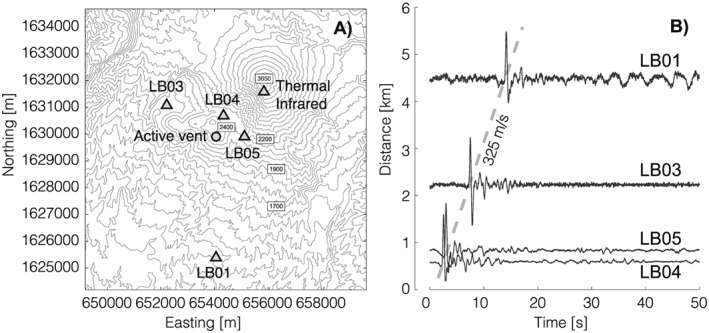
(a) Map showing the infrasound network and thermal infrared observation point at Santiaguito. (b) Unfiltered, individually normalized, infrasound waveforms recorded across the network showing a propagation velocity of 325 m/s.

Santiaguito is a complex of dacitic lava domes that formed within the collapse scar of the 1902 eruption of Santa Maria volcano, one of the largest in the twentieth century [*Bennett et al.*, 1992]. The ongoing eruption of Santiaguito began in 1922 [*Bluth and Rose*, 2004], with present activity concentrated at the eastern Caliente vent (Figure 1a). Eruptive activity consists of continuous extrusion of blocky lava flows interspersed by frequent gas‐and‐ash explosions, accompanied by occasional collapses and short runout pyroclastic flows. Explosions throughout the past decade have been of small to moderate size and have produced volatile‐rich plumes with relatively minor proportions of ash [*Yamamoto et al.*, 2008]. Recent petrological and geophysical analyses suggest that these small gas‐and‐ash explosions are not triggered by typical magmatic fragmentation but rather through sacrificial fragmentation that occurs along fault zones due to shear‐induced thermal vesiculation [*Lavallée et al.*, 2015].

## 3 Data and Methods

In November 2014 we deployed a network of four iTem prs100 [*Delle Donne and Ripepe*, 2012] infrasound sensors (flat response between 0.01 and 100 Hz, sensitivity 25 mV/Pa, ±100 Pa full scale, and self‐noise −52 dB rel. 1 Pa) at Santiaguito, all within 5 km of the active Caliente vent (Figure 1a). During the initial deployment we also gathered thermal infrared (TIR) imagery of the explosive activity from the summit of Santa Maria, which overlooks the Caliente vent providing a unique observation point at Santiaguito (Figure 1a). The infrasound waveforms recorded during November–December 2014 were characterized by relatively low amplitudes (0.7–2 Pa at a distance of 4.5 km from the vent), had durations of between 5 and 10 s, and included either one or multiple pulses. Unlike previous observations by *Johnson and Lees* [2010], we recorded infrasound signals lacking significant coda phases, and waveform coherency across the network was high (Figure 1b). For this study we used acoustic signals from 61 explosions during the period 29 November to 5 December 2014, when all infrasound sensors were operational. We also analyzed TIR imagery gathered during an explosion on 30 November 2014, with a FLIR T450sc infrared camera equipped with a 30 mm lens (FOV: 15 × 11.25, IFOV: 0.82 mrad) calibrated in the relevant temperature range, and considering atmospheric conditions at Santiaguito.

### 3.1 Acoustic Multipole Source Inversion

We applied the acoustic multipole source inversion method of *Kim et al.* [2012] in order to assess the time history of mass eruption rate at the vent (monopole source term) and the azimuth and strength of a horizontal force (dipole source term) to represent directivity effects within the crater region of the Caliente vent. Following the formalism of *Kim et al.* [2012], a discrete time series of acoustic pressures radiated by a source consisting of a monopole and a horizontal dipole can be written as

(1) $$p_{i}^{k}=\frac{1}{2\pi r_{i}}\left[m_{1}^{k}+\frac{x_{i}}{cr_{i}}m_{2}^{k}+\frac{y_{i}}{cr_{i}}m_{3}^{k}\right]$$

where $p_{i}^{k}$ is the *k*th element of the time series recorded at the *i*th station, *c* is the atmospheric sound speed, and *r*_*i*_ is the distance between the source and the *i*th receiver. The vector **m** ^*k*^ is

(2) $${\text{m}}^{k}=\left[m_{1}^{k},m_{2}^{k},m_{3}^{k}\right]=\left[\dot{S}(t_{0}+\mathrm{k\Delta t}),\dot{F_{x}}(t_{0}+\mathrm{k\Delta t}),\dot{F_{y}}(t_{0}+\mathrm{k\Delta t})\right]$$

where *S* is the strength of the monopole source term (i.e., the mass eruption rate), measured in kg/s, and *F*_*x*_ and *F*_*y*_ are the horizontal components of the dipole force, measured in kg/ms^2^. The system of linear equations in (1) can be rewritten in matrix form as

(3) $${\text{P}}^{k}=\text{G}{\text{m}}^{k}$$

where **P** ^*k*^ is a vector of sampled acoustic pressures at each station. Inversion of equation (3) for **m** ^*k*^ allows assessing monopole and dipole strengths with the only additional requirement that the number of stations must be greater than three. *Kim et al.* [2012] suggested, based on synthetic tests, that the best results for an inversion are obtained from networks of at least three sensors evenly distributed around the source (i.e., with an azimuthal gap of 120°). Our network at Santiaguito satisfies these requirements reasonably, with an azimuthal gap of about 159°. This is the smallest gap that could be achieved given restrictions to access in the western and southwestern sectors of the volcano (Figure 1a).

For the inversion, only high signal‐to‐noise ratio waveforms associated with confirmed explosions and atmospheric propagation of a plume were selected. The inversion was performed over 5 s signal windows including the onset of each event. Linear piecewise detrending was applied to all signals according to the Finite Window Zero Pressure Zero Flux correction proposed by *Johnson and Miller* [2014] in order to mitigate effects of instrument drift. Waveforms were filtered between 0.1 and 4 Hz (2‐pole, Butterworth filter) to include the dominant part of the energy spectrum while mitigating the effects of high‐frequency noise on the stability of the inversion. We used an atmospheric sound velocity of 325 m/s as measured from the moveout of infrasound arrivals across the network (Figure 1b).

### 3.2 Analysis of Thermal Infrared Imagery

TIR video footage was analyzed following the workflow of *Valade et al.* [2014], which allows tracking of ascending volcanic plumes. Thermal images were corrected and transformed from units of pixels to metric units, considering the focal length of the lens, the vertical field of view, the instantaneous field of view, and the distance and inclination between vent and the observation point (the reader may refer to *Valade et al.* [2014] for additional details on these procedures). Following transformation of all TIR video frames to a vent‐centered system of coordinates, and assuming symmetrical plume growth about a vertical axis, a number of eruption parameters were measured, including plume ascent velocity and volume throughout the duration of the eruptive event.

## 4 Results

**Figure 2:**
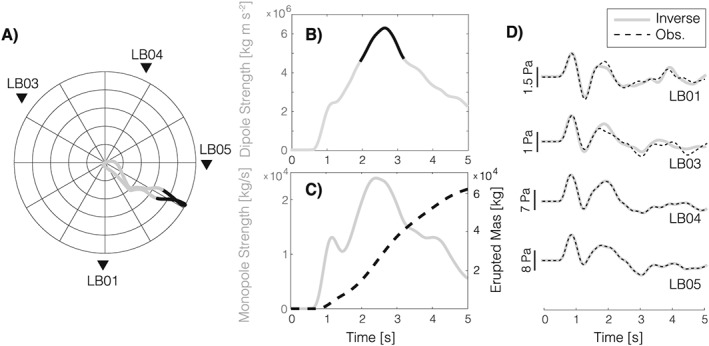
Acoustic multipole source inversion for an explosion at Santiaguito on 30 November 2014 01:09:14 UTC: (a) dipole azimuth over the source time function, (b) dipole strength, (c) monopole strength, and (d) fit between synthetic and observed waveforms.

**Figure 3:**
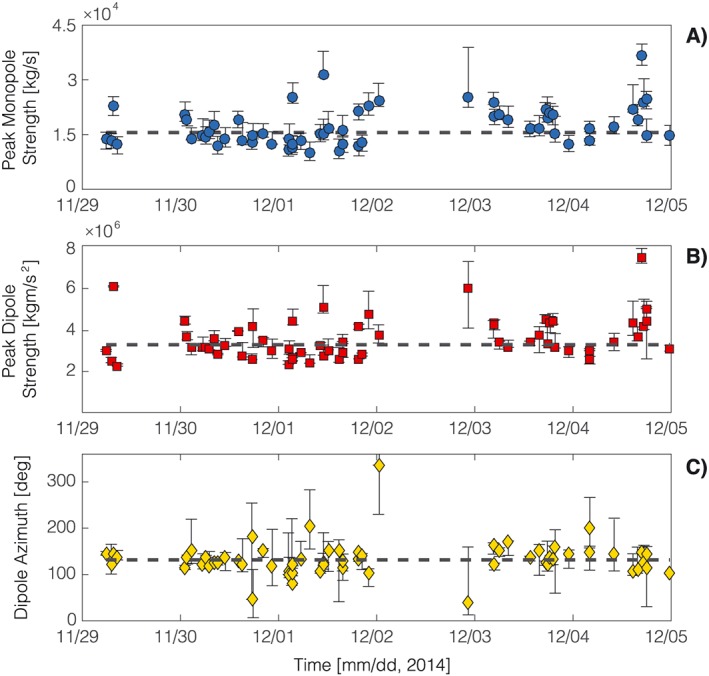
Results of acoustic multipole source inversion for 61 explosions recorded at Santiaguito, between 29 November and 5 December 2014. (a) peak monopole strength, (b) peak dipole strength, and (c) dipole azimuth. Confidence bars reflect the range of values obtained from inversions for all possible combinations of three stations in the network (see text in the manuscript). The dashed line represents the median value of measurements.

We performed multipole source inversion for 61 explosions recorded at Santiaguito during 29 November to 5 December 2014. Results from waveform inversions are illustrated in Figure 2 for an explosion recorded on 30 November at 01:09:14 UTC. The inversion produced a good fit between synthetic signals and observations (Figure 2d). As a measure of fit quality, we calculated variance reduction for the ensemble of *N* waveforms:

(4) $$\mathrm{VR}=\frac{1}{N}\overset{N}{\underset{i=1}{\sum }}\left[1-\frac{\int {\left(d_{i}-s_{i}\right)}^{2}}{\int {\left(d_{i}\right)}^{2}}\right]\cdot 100$$

where *s*_*i*_ and *d*_*i*_ are the *i*th synthetic and data, respectively, and the integrals are performed over the duration of the signals. For the explosion in Figure 2 we obtained VR = 92.3%. The most obvious, yet minor, mismatch between data and synthetics is seen at stations LB01 and LB03 (Figure 2d), later in the waveform, past the well‐fit initial pulse. This was consistently observed for all 61 events analyzed, likely due to the location of stations LB01 and LB03 at comparatively larger distances from the vent. It has been shown that propagation of the acoustic wavefield over terrain with variable topography may result in diffraction, attenuation, or focusing effects beyond the predictions of geometrical spreading [*Lacanna et al.*, 2014]. Recent studies have begun tackling the influence of variable topography and atmospheric conditions on the acoustic wavefield [*Lacanna et al.*, 2014; *Kim et al.*, 2015] suggesting that numerical Green's functions hold great promise for integrating these complex phenomena in infrasound propagation studies and waveform inversion. Here, however, the good agreement between synthetics and data confirms that the effects of topography, where present, are comparatively small. This can be attributed to the location of the sensors that, with the only exception of LB03, benefit from unobstructed source‐receiver paths (supporting information Figure 1S). The dominant direction of the dipole azimuth for this event is 120° (Figure 2a), suggesting ESE directivity. Figure 3 illustrates the results of multipole source inversions for all 61 events analyzed; peak monopole and dipole strength, and dipole azimuth, are stable throughout the study period, reflecting the regular character of activity at Santiaguito. In order to assess the stability of our results, and the sensitivity of the method to changes in network geometry, we conducted a test performing waveform inversion for all explosions using all possible combinations of only three stations. Results are illustrated by the confidence bars in Figure 3, which represent the range of values obtained from all inversions for each of the parameters evaluated. It is worth noting that the largest deviation from the results using data from the entire network was consistently observed removing station LB01 from the inversion. This was expected as removal of station LB01 introduces the largest azimuthal gap among all possible three‐station network configurations.

**Figure 4:**
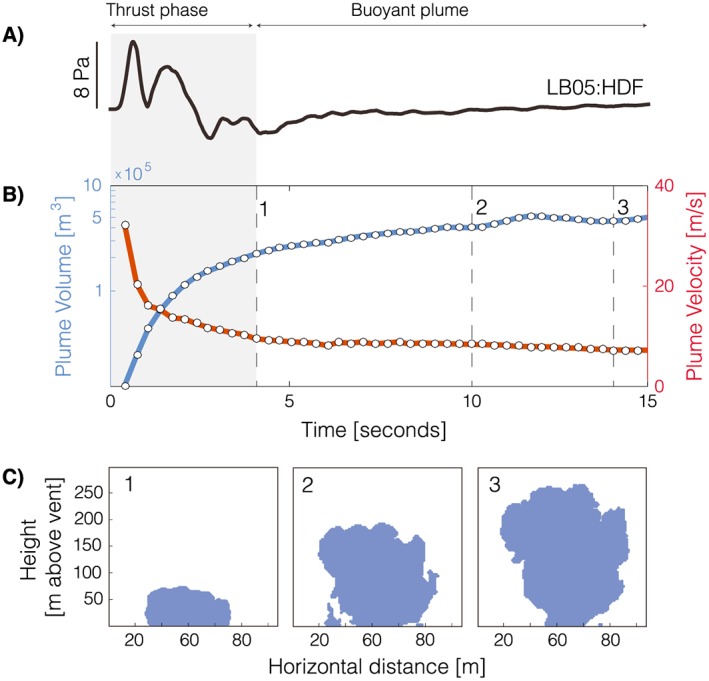
Time synchronized infrasound and thermal infrared (TIR) data for the explosion in Figure 2. (a) Infrasound signal recorded at station LB05, (b) plume volume and plume velocity from the analysis of TIR imagery, and (c) cross‐sectional view of the plume from the analysis of TIR. Frames 1–3 correspond to times 1–3 in Figure 4b.

Results of the analysis of TIR data recorded on 30 November, for the explosion at 01:09:14 UTC, are illustrated in Figure 4. Figures 4a and 4b display infrasound recorded at station LB05, the time history of plume volume, and its ascent velocity, respectively; Figure 4c illustrates the temporal evolution of the cross section (side view) of the rising plume. The time series of plume ascent velocity (measured at the plume front) allows identifying the transition from an early, short‐duration, thrust phase dominated by a momentum‐driven volcanic jet, to a buoyant regime where the morphology of the plume was controlled by entrapment of surrounding, colder, atmospheric air [*Patrick*, 2007; *Marchetti et al.*, 2009; *Delle Donne and Ripepe*, 2012]. The timing of this transition, which corresponds to the point when the plume ascent velocity stabilizes around values <10 m/s in Figure 4c (point 1 at about 4.2 s), can be used to constrain the cumulative volume erupted before engulfment of atmospheric air became dominant. From the data presented in Figure 4b, we estimated this volume to be 2.2·10^5^ m^3^. We suggest that this value provides a reasonable first‐order approximation for the cumulative volume of ash and gas injected from the volcanic vent into the atmosphere during the explosion. Finally, the monopole strength function (Figure 2c) was integrated over the duration of the thrust phase to provide an estimate of the cumulative erupted mass of 5.9·10^4^ kg. Taken together, these values suggest a bulk density of the plume, during the initial thrust phase, of 0.27 kg/m^3^.

## 5 Discussion and Conclusive Remarks

An integrated, multidisciplinary, approach can help to improve our understanding of the mechanisms of volcanic explosions and the dynamics of plume rise. Here we have shown that the strength of acoustic sources, and the directivity of the associated wavefield, during volcanic explosions can be estimated by inversion of infrasound waveforms, and the erupted volume can be retrieved from analysis of TIR or visual imagery. The strength of the acoustic monopole source time function presented here, in Figure 2c, is consistent with previous measurements of eruption rates at Santiaguito [*Cerminara et al.*, 2015] based on plume modeling and inversion of TIR data. The strength of the dipole function at Santiaguito (Figure 2b) is about 2 orders of magnitude smaller than values reported for larger Vulcanian explosions at Tunghuraua volcano [*Kim et al.*, 2012] and Strombolian explosions at Mount Erebus [*Johnson et al.*, 2008]. However, we had anticipated a comparatively weaker dipole as peak infrasound amplitudes at Santiaguito are 2 orders of magnitude smaller than those recorded at Tunghuraua and Mount Erebus at similar distances from the vent. We stress that this is not equivalent to a weak (negligible) dipole in relation to the strength of the monopole source term. On the contrary, the dipole term plays a crucial role in accounting for the directivity of infrasound although the origin of the directional nature the acoustic wavefield in the source region cannot be fully resolved. In the future, numerical modeling needs to be integrated within the acoustic inversion workflow to evaluate the influence of factors such as near‐source topography (e.g., the geometry of the crater wall) on volcanic infrasound. In order to confirm the importance of the dipole term for our results, we attempted to estimate mass eruption rate via direct integration of the infrasound time series (i.e., a monopole‐only source) recorded at each station in the network following *Johnson and Miller* [2014]. We observed significant variability between stations and could not obtain stable results, with LB03 showing the largest deviation from the multipole inversion.

Acoustic multipole source inversion relies on the assumption that the source can be considered acoustically compact [*Rossing*, 2016]; that is, its largest dimension is small relative to the dominant wavelength of the signal. Previous studies suggested that infrasound at Santiaguito may be generated by uplift of the entire dome (a roughly circular structure, about 200 m in diameter), before the onset of explosions, a source that would be inherently noncompact [*Johnson and Lees*, 2010]. Our recent observations show that the initial compression in the infrasound signals coincides with the onset of the plume in the visual and TIR images (considering infrasound propagating at 325 m/s and GPS time synchronization within 1/30 s between TIR and infrasound). Video footage from recent explosions at Santiaguito also revealed that they may initiate at subparallel linear faults near the center of the dome [*von Aulock et al.*, 2016], rather than along arcuate or ring faults along its periphery as it was commonplace in the past. The typical dimension of these linear fissures is of the order of 20–30 m, and thus, they can be considered acoustically compact with respect to the dominant wavelength of infrasound at Santiaguito (350–650 m).

Analysis of TIR imagery allowed identifying the timing of the transition between an early, short‐duration, gas thrust phase and a buoyant plume. We measured both the cumulative erupted volume and mass at this point, which we consider reliable proxies for the total amount of material injected from the vent into the atmosphere. They possibly represent a slight underestimate as ash and gas venting may continue, albeit with lower intensity, during the buoyant phase. *Bluth and Rose* [2004] had previously reported the absence of a gas thrust phase at Santiaguito and suggested that plume rise was controlled by buoyancy at the source. Their inference was supported by the observation that past eruptive activity originated, predominantly, at annular cracks along the periphery of the dome. While our data confirm that late plume rise at Santiaguito is controlled by buoyancy, they also demonstrate that during recent explosions, an early thrust phase was present. This observation is consistent with eruptive plumes sourced at compact, elongated, fractures such as those recently identified at Santiaguito [*von Aulock et al.*, 2016].

Finally, we estimated the bulk density of the initial plume, *ρ*_plume_=0.27 kg/m^3^, at the time of the gas thrust‐buoyancy transition. Additionally, we collected samples of volcanic ash and measured its density, *ρ*_ash_=2650 kg/m^3^, using He pycnometry. These data can be used to estimate the relative volume fractions of ash and gas erupted during the explosion. We considered that the main volatile phase in the plume is water vapor [*Yamamoto et al.*, 2008] with density *ρ*_gas_=0.15–0.21 kg/m^3^ (at eruption temperatures of 1000–1400 K and decompression to atmospheric pressure). The volume fraction of ash in a plume *ϕ* can, then, be calculated from the simple equation:

(5) $$\phi =\frac{\rho_{\text{plume}}-\rho_{\text{gas}}}{\rho_{\text{ash}}-\rho_{\text{gas}}}$$

For the range of plume, ash, and gas densities considered in our study, *ϕ* = 2.3–4.5·10^−5^, which is about 1 order of magnitude lower than previous, independent, estimates by *Yamamoto et al.* [2008]. We note that these results are sensitive to the particular choice of ash density. If ash density is considered to be 650 kg/m^3^, as in *Yamamoto et al.* [2008], our estimates of *ϕ* would fall in the range 9.2·10^−5^–1.8·10^−4^, closer to the values presented by these authors.

In conclusion, we have demonstrated that combined analyses of infrasound, TIR, visual, and field data can shed light on the dynamics of explosions at lava dome volcanoes. We have investigated moderate gas‐and‐ash explosions at the Santiaguito lava dome complex and observed that eruptive plumes exhibit transitions between an early, short‐duration, thrust phase and later buoyant rise. We assessed the relative volume fractions of gas and ash erupted during a single explosion and demonstrated that plumes from small‐to‐moderate size events at Santiaguito are extremely gas rich. The minor volume fraction of ash in the plume suggests that these events may not involve significant fragmentation of magma in the conduit and concurs with the recent suggestion that local sacrificial fragmentation along fault zones, due to shear‐induced thermal vesiculation, may be at the origin of such events [*Lavallée et al.*, 2015].

We surmise that the combined use of infrasound and TIR, or visual, imagery holds great promise for assessment of mass eruption rates and volcanic plume rise, with potential applications in near real time. We envision that numerical Green's functions will be included in future developments of infrasound waveform inversion schemes in order to account for the effects of near‐vent geometry, local topography, and variable atmospheric conditions on volcanic infrasound. For larger eruptions, mass eruption rate is a key input parameter in models of atmospheric plume rise and ash dispersal, and thus, its rapid assessment is crucial to mitigating the risks associated with volcanic eruptions.

## Supporting information



Supporting Information S1
