# Supplementary material for: Characterization of moderate ash‐and‐gas explosions at Santiaguito volcano, Guatemala, from infrasound waveform inversion and thermal infrared measurements
Source: Geophys Res Lett. 2016 Jun 27;43(12):6220–7. doi: 10.1002/2016GL069098 (PMC5405577; doi:10.1002/2016GL069098)
Supplement: Supplementary file 1 — Supporting Information S1 [file GRL-43-6220-s001.doc]

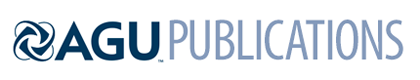


*Geophysical Research Letters*

Supporting Information for

Characterization of moderate ash-and-gas explosions at Santiaguito volcano, Guatemala, from infrasound waveform inversion and thermal infrared measurements.

S. De Angelis^(1)^, O.D. Lamb^(1)^, A. Lamur^(1)^, A.J. Hornby^(1)^, F.W. von Aulock^(1)^, G. Chigna^(2)^, Y. Lavallée ^(1)^ and A. Rietbrock^(1)^

^(1)^ School of Ocean and Environmental Sciences, University of Liverpool, UK

^(2)^ Instituto Nacional de Sismologıa, Vulcanologıa, Meteorologıa e Hidrologıa (INSIVUMEH), Guatemala

**Contents of this file**

Figure S1

Figure S1. A) through D): Source-receiver paths for infrasound stations at the Santiaguito lava dome, Guatemala. The panels show that all locations, with exception of LB03, benefit from an unobstructed view of the active volcanic vent.
